# In-Car Environment Control Using an SSVEP-Based Brain-Computer Interface with Visual Stimuli Presented on Head-Up Display: Performance Comparison with a Button-Press Interface

**DOI:** 10.3390/s24020545

**Published:** 2024-01-15

**Authors:** Seonghun Park, Minsu Kim, Hyerin Nam, Jinuk Kwon, Chang-Hwan Im

**Affiliations:** 1 Department of Electronic Engineering, Hanyang University, Seoul 04763, Republic of Korea; s.park7532@gmail.com (S.P.); kwom2000@hanyang.ac.kr (J.K.); 2 Department of Artificial Intelligence, Hanyang University, Seoul 04763, Republic of Korea; minsukim207@gmail.com (M.K.); leanonlyn@gmail.com (H.N.); 3 Department of Biomedical Engineering, Hanyang University, Seoul 04763, Republic of Korea

**Keywords:** advanced driver assistance, brain-computer interfaces (BCIs), head-up display (HUD), safe driving, steady-state visual evoked potential (SSVEP)

## Abstract

Controlling the in-car environment, including temperature and ventilation, is necessary for a comfortable driving experience. However, it often distracts the driver’s attention, potentially causing critical car accidents. In the present study, we implemented an in-car environment control system utilizing a brain-computer interface (BCI) based on steady-state visual evoked potential (SSVEP). In the experiment, four visual stimuli were displayed on a laboratory-made head-up display (HUD). This allowed the participants to control the in-car environment by simply staring at a target visual stimulus, i.e., without pressing a button or averting their eyes from the front. The driving performances in two realistic driving tests—obstacle avoidance and car-following tests—were then compared between the manual control condition and SSVEP-BCI control condition using a driving simulator. In the obstacle avoidance driving test, where participants needed to stop the car when obstacles suddenly appeared, the participants showed significantly shorter response time (1.42 ± 0.26 s) in the SSVEP-BCI control condition than in the manual control condition (1.79 ± 0.27 s). No-response rate, defined as the ratio of obstacles that the participants did not react to, was also significantly lower in the SSVEP-BCI control condition (4.6 ± 14.7%) than in the manual control condition (20.5 ± 25.2%). In the car-following driving test, where the participants were instructed to follow a preceding car that runs at a sinusoidally changing speed, the participants showed significantly lower speed difference with the preceding car in the SSVEP-BCI control condition (15.65 ± 7.04 km/h) than in the manual control condition (19.54 ± 11.51 km/h). The in-car environment control system using SSVEP-based BCI showed a possibility that might contribute to safer driving by keeping the driver’s focus on the front and thereby enhancing the overall driving performance.

## 1. Introduction

It is believed that up to 14% of all traffic accidents are caused by distracted driving, making it one of the most prevalent causes of collisions [1,2,3]. In addition, considering that distracted driving is underreported because of the difficulty of its verification in practice, the actual contribution of distracted driving to traffic accidents is likely to be underestimated [3,4]. However, current methods to control in-car environments, such as ventilation and temperature, necessarily cause distracted driving because they require the drivers to switch their attention from the front to the control buttons or a touch screen.

Brain-computer interface (BCI) is a technology that allows its users to directly control external devices using their brain activity [5,6,7], which might be a plausible solution to the above-mentioned issue. Since the first proposal of the BCI concept based on electroencephalography (EEG) [5], a variety of BCI applications have been developed to help paralyzed individuals who have difficulty controlling external devices and communicating with the external environment [8,9,10]. Recently, BCI technologies have been actively employed to augment the capabilities of healthy people in various practical applications [11,12,13,14], including vehicular applications to assist in safe driving [15,16]. For example, Lin et al. [17] proposed a real-time drowsiness detection system based on a BCI to warn drivers when they feel drowsy. Similarly, Wang et al. [18] proposed a BCI-based system for detecting mental fatigue during driving.

While various BCI applications are being actively developed to detect driver alertness and drowsiness, the use of BCI technologies to control in-car environments has rarely been discussed. For example, an in-vehicle control system using motor-imagery (MI)-based BCI was proposed by Cernea et al. [19]; however, the system was not suitable for actual driving situations because MI-based BCIs generally require a high level of cognitive load and long calibration time. To address this issue, Bellotti et al. proposed a vehicle infotainment control system using a visual P300-based BCI that employed intuitive visual stimuli that allowed the driver to select command icons simply by staring at the flickering visual stimulus [20]. However, their study employed a 5-inch LCD screen as a rendering device to present visual stimuli, which made the drivers change their gaze from the front windshield to the dashboard of the car to use the system, which also caused driver distraction while driving. More importantly, the classification accuracy of their system did not reach a practical level. It was as low as 53% in the six-class classification (chance level = 16%).

To address these limitations, we employed a BCI system based on steady-state visual evoked potential (SSVEP), which is a periodic brain response elicited by a flickering or reversing visual stimulus at a certain frequency [21]. SSVEP-based BCIs have the following advantages over P300-based BCIs: (i) they are more robust to noise, (ii) they require lesser data and are hence faster, and (iii) they do not require any training procedure [22,23]. Due to such advantages, the choice was made to employ SSVEP-based BCI over alternative BCI methods, including P300- or MI-based BCIs. Furthermore, SSVEP-based BCIs are intuitive to use because SSVEP responses can be induced simply by staring at target visual stimuli. Additionally, in this study, visual stimuli were presented on a head-up display (HUD) so that the drivers could control the in-car environment while driving without changing their gaze away from the front windshield.

In this study, we investigated the performance of the proposed BCI-based in-car environment control system in a more realistic driving environment by using a driving simulator, and the performance of the proposed BCI system was compared with that of the conventional “button-press” interface. The participants performed two different driving tasks: the “avoiding obstacles” task and the “following a preceding car” task. Both tasks were conducted in a realistic urban and highway environment by using a driving simulator. Then, the driving performances in the “BCI control” condition were quantitatively compared with those in the “manual control” condition with buttons on the dashboard. The experiments were conducted in real-time, and the control results were immediately provided to the drivers with auditory feedback.

## 2. Related Works

Since the SSVEP-based BCI holds many advantages over other BCI modalities, SSVEP has been used on the system to control external devices. Müller et al. [24] introduced the SSVEP-BCI system designed to command a robotic wheelchair by discriminating four classes at a rate of once per second. The authors demonstrated the capability of their BCI system to operate a robotic wheelchair in real-time, achieving an average accuracy of 83%. Additionally, Ke et al. [25] implemented an online SSVEP-BCI system in an augmented reality environment. The performance of their system was evaluated through a cue-guided target selection task and a robotic arm control task. The results showed an average accuracy of 87.8% and average information transfer rates (ITR) of 64.88 bits/min for the cue-guided target selection task, and 75.5% and 45.57 bits/min for the robotic arm control task, demonstrating the potential of a wearable SSVEP-BCI system in augmented reality environment.

The feasibility of using HUD for displaying visual stimuli was investigated in previous studies [26,27]. In Bi et al.’s study [26], the authors investigated whether the SSVEP-based BCI with visual stimuli presented on HUD can be used to control a vehicle. In their next work [27], the authors also investigated the feasibility of using the SSVEP-based BCI for controlling an in-car device. They employed a single-target SSVEP stimulus to turn on and off a device every 30 s during simulated driving. In their experiments, only four participants conducted two primary driving tasks including lane keeping and obstacle avoiding; however, they did not compare the performance of the proposed interface with that of the conventional “button-press” interface. Moreover, since their study was merely a preliminary study to show the feasibility of using HUD for controlling in-car devices, their simulation settings were not realistic enough.

Safety is one of the most important factors in designing vehicles. Kern and Schmidt [28] discussed that the increased in-car functionalities may diminish the driver’s concentration on driving, highlighting the importance of minimizing distractions posed by in-car functionalities. Ng et al. [29] conducted a comparative analysis of various input control types for in-car interactions, including pressure buttons, dials, direct touch, and touch buttons. Their investigation involved assessing the glance count on the touchscreen for each input type, revealing the lowest count of 35 times for direct touch and the highest count of 121 times for pressure buttons. The elevated glance counts during driving would increase the potential for driver distraction. However, the proposed SSVEP-BCI system obviates the need for diverting the driver’s gaze away from the front, thereby promoting safer driving.

## 3. Materials and Methods

### 3.1. Subjects

A total of 32 healthy adults (17 males and 15 females, aged 24.5 ± 3.0 years) volunteered to participate in the experiments; however, 10 participants were screened out because no spectral peak was identified at any SSVEP stimulation frequency during an 8-trial screening session in which visual stimuli were provided before the driving experiment. Therefore, 22 out of 32 participants (13 males and 9 females, aged 24.9 ± 3.4 years) took part in the driving session, and their EEG data were considered in further analyses. This so-called “BCI-illiteracy” is a well-known issue for all types of EEG-based BCIs [30]. All participants had normal or corrected-to-normal vision and possessed a valid driver’s license. All participants provided written consent after being informed of the experimental details. This study was approved by the institutional review board (IRB) of Hanyang University, Republic of Korea (IRB No. HYI-14-167-13) according to the Declaration of Helsinki.

### 3.2. Simulated Driving Environment

**Figure 1:**
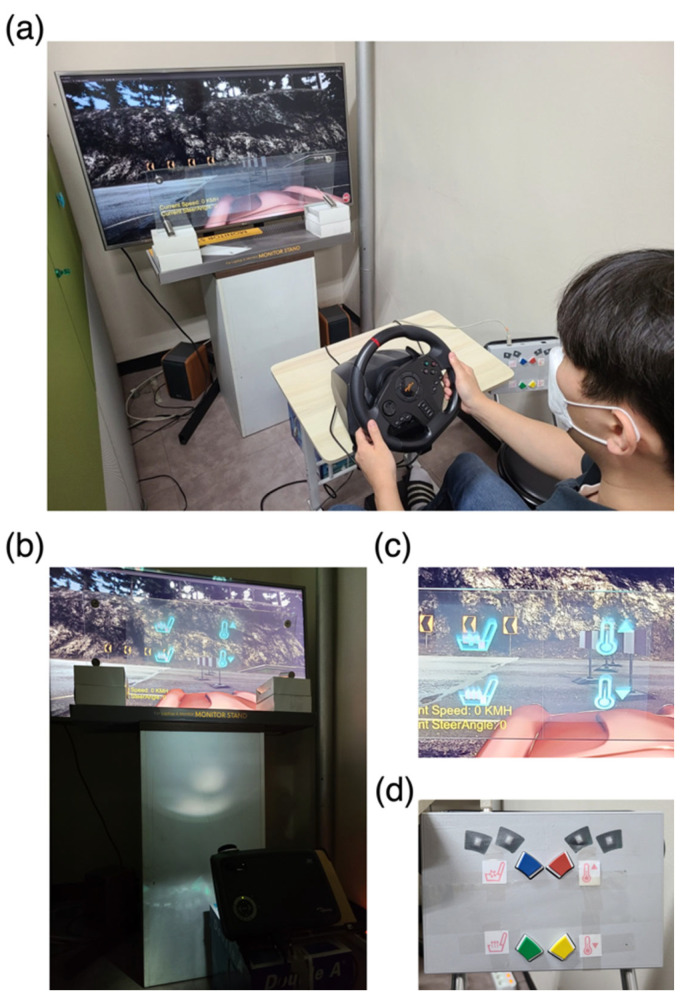
The real pictures of the experimental environment: (**a**) The simulated driving environment. The participants could control the car using a gaming wheel and pedals. (**b**) The HUD setting was employed in the experiment. The projector on the floor projected the visual stimulus on a light reflection film attached to a glass panel in front of the TV, thereby enabling the participants to see the TV screen through the HUD. (**c**) The visual stimulus presented on HUD. (**d**) The configuration of buttons for manual control.

We built a bespoke driving map on the AirSim open-source platform [31], version 1.3.1, using Unity 3D, version 2021.3.8f1 (Unity Software Inc., San Francisco, CA, USA), and a 49-inch TV (LG Electronics Inc., Seoul, Republic of Korea) was used to present the simulated driving environment (Figure 1a). A commercial gaming wheel and pedals (PXN-V900, Shenzhen PXN Electronics Technology Co., Ltd., Shenzhen, China) were used to operate the vehicle. A control panel (RB-830, Cedrus Corporation, San Pedro, CA, USA) was used to simulate the dashboard (Figure 1d). The participants sat on a comfortable armchair, where the steering wheel, pedals, and control panel were positioned similarly to their locations in an actual car.

### 3.3. Visual Stimulus Presented on HUD

To elicit SSVEP responses, we employed a grow/shrink stimulus (GSS), which is a star-shaped stimulus that flickers and varies in size concurrently at a constant frequency, as it has been reported that SSVEP-BCIs with GSS improve classification accuracy compared to those with conventional stimuli [10]. Additionally, we used a GSS with a red/blue-colored checkerboard pattern to further enhance the SSVEP response [32]. Four visual stimuli flickering at 7.5, 8.57, 10, and 12 Hz were employed, each assigned to four commands to control the in-car environments (Table 1). Each command has been chosen for its frequent use in real-world vehicular scenarios. Given the simulated environment, participants received auditory feedback in place of the actual execution of each function. For some participants, based on the results of the preliminary screening session, a visual stimulus flickering at 6 Hz was alternatively employed to replace the 12 Hz stimulus.

Then, using a digital light processing projector (EP728, Optoma, New Taipei City, Taiwan), the visual stimulus was presented on a light reflection film (440 mm × 300 mm) affixed to a tempered glass panel that was 700 mm × 240 mm × 8 mm in size, as shown in Figure 1b. Furthermore, as shown in Figure 1c, the simulated driving environment was clearly visible through the HUD while visual stimuli were presented on the HUD.

### 3.4. Experimental Paradigm

**Figure 2:**
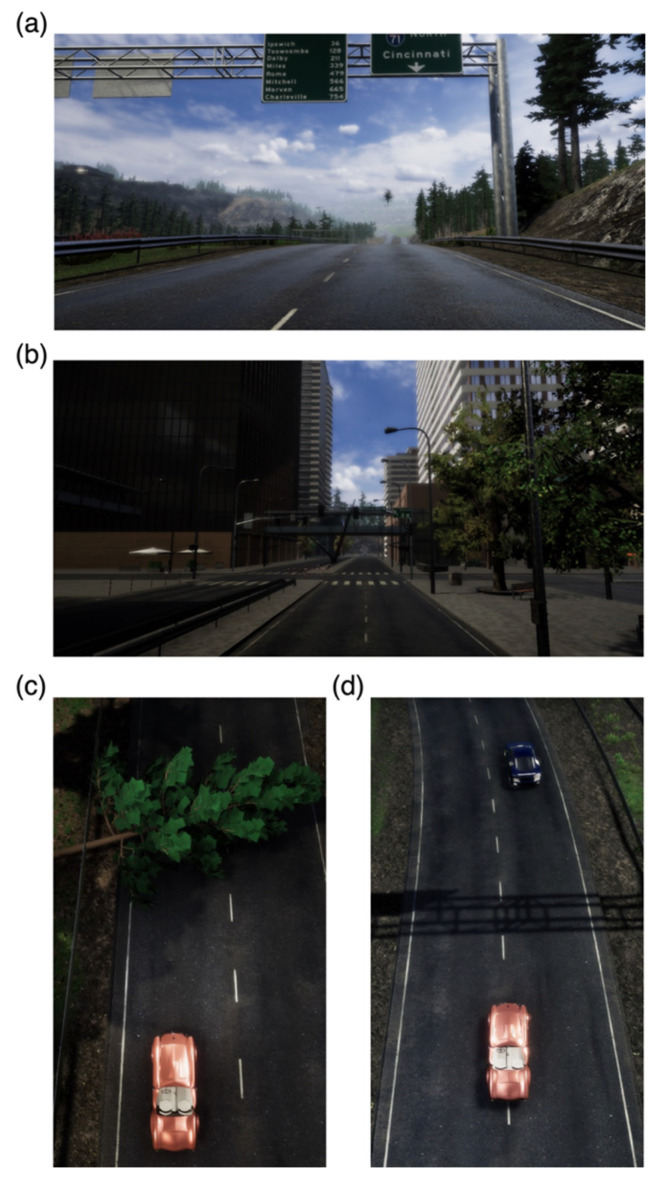
The screenshots of the simulated driving environment. (**a**) The highway course with a speed limit of 100 km/h; (**b**) The city course with a speed limit of 60 km/h; (**c**) An example of obstacle employed in obstacle avoidance driving test; (**d**) The participant’s car (red) and the preceding car (blue).

The participants were instructed to drive a simulated car following driving courses that consisted of a highway and an urban road. The speed limit on the course was set to 100 km/h on the highway (Figure 2a) and 60 km/h on the urban road (Figure 2b). The participants had to drive their car at a speed as close to the speed limit as possible while ensuring that it was not exceeded. The time required to drive the entire course was approximately 3 min, as it took approximately 1 min 30 s each to complete driving over the highway and urban road.

Two driving tests were included in the experiment: (1) an obstacle avoidance test and (2) a car-following test. In the obstacle avoidance test, four obstacles unexpectedly appeared on the driving course, and the participants were required to stop their vehicle as soon as possible to avoid a collision (Figure 2c). In the car-following test, participants had to follow a preceding vehicle that was driven at a sinusoidal speed profile (Figure 2d). They were instructed to maintain a constant distance from the preceding car and to travel along the centerline of the lane as closely as possible. The speed limit was not applied in the car-following test.

In addition, each driving test was composed of two in-car environment control conditions: (1) manual control and (2) SSVEP-BCI control. In the manual control condition, the participants were instructed to control the in-car environment by manually pressing a designated button (Figure 1d), whereas, in the SSVEP-BCI control condition, they were required to control the in-car environment by simply staring at a designated visual stimulus presented on the HUD (see Figure 1c). The obstacle avoidance test was performed before the car-following test. Both control conditions for each driving test were conducted in random order. Consequently, each participant conducted four driving tests {(two driving tests) × (two control conditions)}. In each run, the participants drove the entire driving course, including the highway and urban road, and the driving course for each run was set to be different to prevent any learning effects.

**Figure 3:**
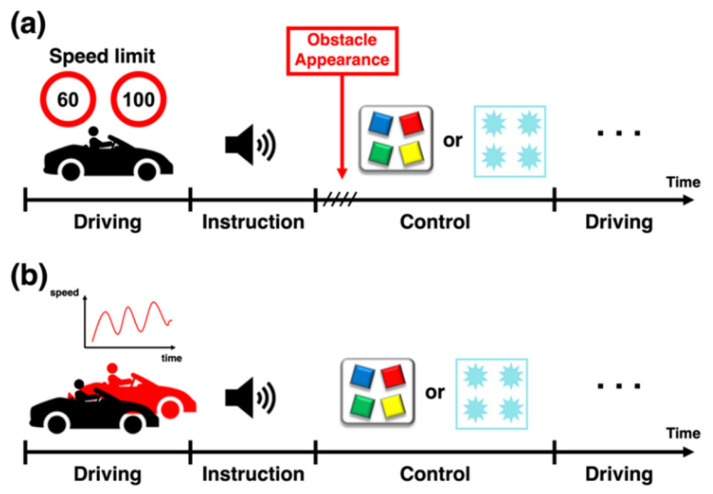
The experimental paradigms for (**a**) obstacle avoidance test and (**b**) car-following driving test. The participants had to drive at a speed within the speed limit in the obstacle avoidance driving test, while they had to follow a preceding car at a constant distance in the car-following driving test. In the control phase, the participants had to execute a designated control command by pressing a button in the manual control condition or staring at a visual stimulus in the SSVEP-BCI control condition.

The participants executed four control commands in each run, immediately after being presented with a guided voice letting them know which command should be executed, for example, “please turn on heated seat”, and a short pure-tone beep sound to notify them that it was time to execute the designated control command. It was confirmed that the auditory instruction did not disturb the driver’s performance. After the instructions were given, the participants executed the designated control command by manually pressing a button and by staring at a visual stimulus in the manual and SSVEP-BCI control conditions, respectively. Specifically, in the SSVEP-BCI control condition, four icons representing each control command were presented on the HUD during the auditory instruction, and four flickering visual stimuli were presented for 2.5 s right after the beep sound was presented. In the obstacle avoidance driving test, an obstacle appeared while the participants controlled the in-car environment. After controlling the car environment either by a manual button press or SSVEP-BCI, the executed control command was provided to the participants in the form of auditory feedback (e.g., “heated seat was turned on”). The experimental paradigms are illustrated in Figure 3. A video clip of the main experiments can be found at https://youtu.be/u-LnJ3NVSEA, accessed on 11 January 2024.

### 3.5. Data Recording and Analysis

Nine electrodes were used to record EEG data at a sampling rate of 2048 Hz (Cz, POz, PO3, PO4, PO7, PO8, Oz, O1, and O2) via a biosignal recording system (ActiveTwo; Biosemi, Amsterdam, The Netherlands). The EEG data were re-referenced to Cz and downsampled to 512 Hz. Then, a Butterworth bandpass filter with 2 and 54 Hz cutoff frequencies was applied to the data. The extension of the multivariate synchronization index (EMSI) algorithm [33] was employed to classify the SSVEP responses, and the EEG data analysis was conducted using a custom real-time software developed using MATLAB 2020b (MathWorks, Inc., Natick, MA, USA). In our software, we applied real-time signal processing and the EMSI algorithm to EEG signals for three seconds starting from when the visual stimuli were presented. The EMSI algorithm calculates the synchronization between the EEG signals and reference signals, which are generated using stimulation frequencies and their harmonics. Then, a target command is determined by identifying the specific frequency that yields the maximum synchronization index. Temporal alignment between the real-time signal processing software, visual stimuli, and the driving environment could be achieved by connecting the computers via serial communication.

The following measures were computed to evaluate the driving performance:

(1) Response time (obstacle avoidance test)

The average time taken to brake the car after the appearance of an obstacle. Note that trials with a response time too short or too long (shorter than 330 ms or longer than 2500 ms) were excluded because they were considered false or missed responses based on a previous study [34].

(2) No response rate (NRR) (obstacle avoidance test)

The ratio of obstacles that the participants did not react to. Note that trials with response times that were too short or long (shorter than 330 ms or longer than 2500 ms) were counted as ‘no response’ trials.

(3) Speed difference (car-following test)

The average of the absolute speed difference between the participant’s car and the preceding car for 3 s after the beep sound [35].

(4) Centerline deviation (car-following test)

The average of the absolute difference between the actual driving route of the participant’s car and the centerline of the lane during the 3 s after the beep sound.

To test the statistical significance, the Wilcoxon signed-rank test was conducted since the Kolmogorov-Smirnov test revealed that the test datasets did not follow a normal distribution. The statistical significance level was set at *p* = 0.05.

## 4. Results

**Figure 4:**
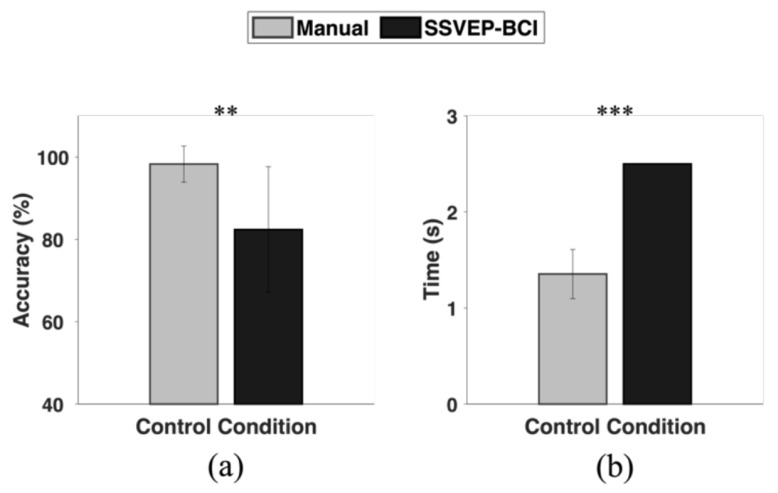
Comparison of the in-car environment control performance between the manual and SSVEP-BCI control conditions: (**a**) control accuracy; (**b**) time duration required to complete a control task (** represents *p* < 0.0005 and *** represents *p* < 0.00005).

The accuracies of in-car environment control in the manual control and SSVEP-BCI control conditions were reported to be 98.3 ± 4.4% and 82.4 ± 15.3%, respectively (Figure 4a). Additionally, the time durations required to control the in-car environment were 1.4 ± 0.26 s and 2.5 s, in the manual and SSVEP-BCI control conditions, respectively (Figure 4b). (Note that the control time in the SSVEP-BCI control condition was fixed at 2.5 s for all participants, whereas it varied in the manual control condition depending on their response times). The Wilcoxon signed-rank test showed significant statistical differences between the two conditions in both accuracy and response time, suggesting that the performance of the current system is not as high as that of the conventional manual control system.

**Figure 5:**
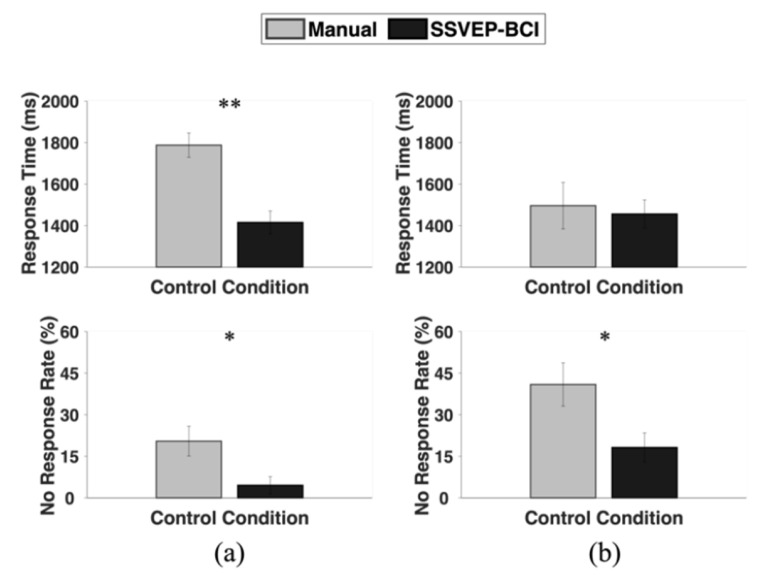
Comparison of the response time and NRR between the manual and SSVEP-BCI control conditions on (**a**) highway; and (**b**) urban course in the obstacle avoidance driving test. The error bars indicate the standard error (* represents *p* < 0.05 and ** represents *p* < 0.0005).

Although the accuracy and control time in the manual control condition were better than those obtained for the SSVEP-BCI control condition, the participants’ driving performance was superior under the SSVEP-BCI control condition compared to its manual equivalent. Figure 5 shows the comparison of the driving performance between the manual and SSVEP-BCI control conditions in the obstacle avoidance driving test. While driving on the highway course, the participants showed significantly shorter response times in the SSVEP-BCI control condition (1.42 ± 0.26 s) than in the manual control condition (1.79 ± 0.27 s) (*p* = 0.00043), while NRR was also significantly lower in the SSVEP-BCI control condition (4.6 ± 14.7%) than in the manual control condition (20.5 ± 25.2%) (*p* = 0.039), as shown in Figure 5a. Meanwhile, NRR on the urban course was also significantly lower in the SSVEP-BCI control condition (18.2 ± 24.6%) than in the manual control condition (40.9 ± 36.6%) (*p* = 0.018), while no statistical significance was observed in the response times between the manual (1.50 ± 0.53 s) and SSVEP-BCI control conditions (1.46 ± 0.32 s) (*p* = 0.81), as shown in Figure 5b.

**Figure 6:**
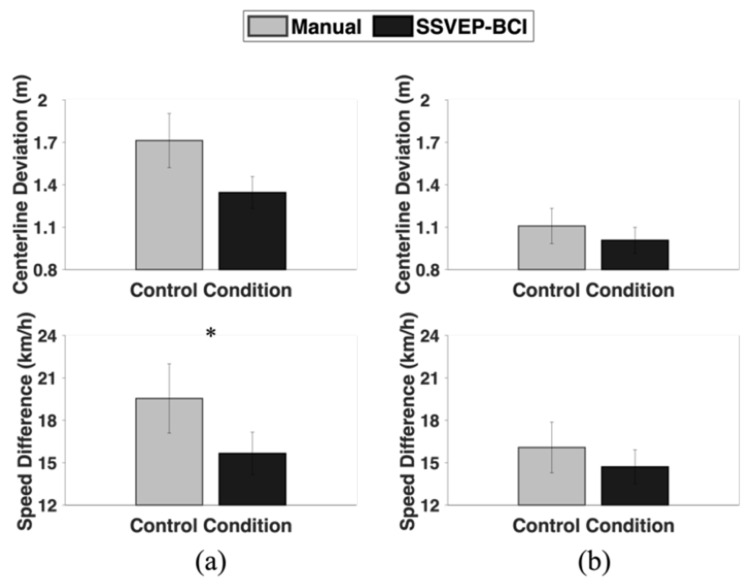
Comparison of the centerline deviation and speed difference between the manual and SSVEP-BCI control conditions for (**a**) highway; and (**b**) urban course in the car-following driving test. The error bars indicate the standard error (* represents *p* < 0.05).

In the car-following driving test with the highway course, the speed difference between the participant’s vehicle and the preceding vehicle was significantly lower in the SSVEP-BCI control condition (15.65 ± 7.04 km/h) than in the manual control condition (19.54 ± 11.51 km/h) (*p* = 0.0495), while the deviation between the centerline and trajectory of the participant’s vehicle was marginally smaller in the SSVEP-BCI control condition (1.35 ± 0.53 m) than in the manual control condition (1.71 ± 0.90 m) (*p* = 0.067; Figure 6a). Meanwhile, on the urban course, no statistical significance was observed in the centerline deviations between the manual (1.11 ± 0.59 m) and SSVEP-BCI control conditions (1.01 ± 0.43 m) (*p* = 0.68), nor the speed differences between the manual (16.08 ± 8.40 km/h) and SSVEP-BCI control conditions (14.71 ± 5.65 km/h) (*p* = 0.61), as shown in Figure 6b.

## 5. Discussion

In the present study, we implemented an in-car environment control system employing an SSVEP-based BCI and visual stimuli displayed on a HUD. We evaluated the feasibility of the proposed system in a realistic simulated driving environment and compared the driving performance under two in-car environment control conditions: SSVEP-BCI and conventional manual (button-press) control conditions. Using the proposed environment control system, the participants could successfully control the in-car environment according to the given instructions and showed better driving performance than in the manual control condition. In the obstacle avoidance driving test, the reaction time was significantly shorter, and the NRR was significantly lower in the SSVEP-BCI control condition than in the manual control condition. Furthermore, in the car-following driving test, the speed difference between the participant’s car and the preceding car was significantly smaller, and the deviation between the directed trajectory (i.e., centerline) and the actual trace of the participant’s vehicle was marginally smaller in the SSVEP-BCI control condition than in the manual control condition. These results imply that the proposed in-car environment control system based on SSVEP-BCI could facilitate safer driving and, consequently, reduce the number of automobile accidents.

Given that the obstacles were designed to appear 1–2 s after the control instructions were presented and the time duration required to complete a control task in the manual control condition was 1.4 on average, it is not surprising that the participants showed higher NRR in manual control condition than in the SSVEP-BCI control condition during the obstacle avoidance driving test. On the other hand, the participants showed a lower NRR in the SSVEP-BCI control condition, which is possibly attributable to that the participants could respond to obstacles faster due to the proposed system’s characteristic that does not require them to change their gaze out of the windshield (TV screen in the experiment). This result is also in accordance with a previous study that reported the drivers violated fewer traffic signals when they controlled the car dashboard using BCI than when they had to control by hand because they could maintain their gaze on the road [19]. However, considering that the number of traffic violations is also in proportion to the level of cognitive load the BCI task demands [19], the BCI tasks to control the in-car environment should maintain an appropriate level of demanding cognitive load. In that sense, the proposed in-car environment control system was designed intuitively to use: Simply by staring at the icon-type visual stimulus, they could control the in-car environment according to their desire.

In the car-following driving test, while no statistical significance was observed in the driving performances between the manual and SSVEP-BCI control conditions in the urban course (Figure 6b), the participants showed significantly lower speed difference in the SSVEP-BCI control condition than in the manual control condition during highway course (Figure 6a). Similarly, in the obstacle avoidance driving test, the participants showed significantly lower response time to obstacles in the SSVEP-BCI control condition than in the manual control condition during the highway course (Figure 5a), while there was no significant difference between them on the urban course (Figure 5b). This implies that the proposed SSVEPBCI-based in-car environment control system can be more effective in highway environments where the speed is relatively high, thereby demanding enhanced responsiveness.

Although the participants showed better driving performances in the urban course than in the highway course (e.g., the centerline deviation was lower on average in the urban course than in the highway course, both in the manual and SSVEP-BCI control condition), NRR in the obstacle avoidance driving test was observed to be higher in the urban course than in the highway course, in contrast to the tendency of the other driving performance measures. This can be due to two factors: (i) since the driving course was designed to start from the highway and finish in the urban course, (i.e., the participants always started the experiment by driving from the highway and then entered the urban course afterward), the participants might be less vigilant when finishing highway and entering the urban course as the speed decreases (100 km/h in the highway to 60 km/h in the urban course); (ii) the four obstacles were designed to be unique to prevent training effect, which might vary the difficulties among the obstacles, affecting the participants’ driving performance. However, it is noteworthy that the participants consistently showed significantly lower NRR in the SSVEP-BCI control condition than in the manual control condition, in both highway and urban courses (Figure 5).

The classification accuracy of the proposed system was 82.4% on average, which was higher than that of a previous study that employed a P300-based BCI [20] but was not high enough to be applied to practical scenarios. Considering that classification accuracies of 90% and higher have been reported in many studies that presented visual stimuli on LCD monitors for SSVEP-based BCIs [36,37], this relatively low classification accuracy is possibly due to the transparent rendering device (i.e., HUD). A previous study that employed a see-through display to present visual stimuli in an SSVEP-based BCI also reported performance degradation [10]. Additionally, the BCI illiteracy rate of 30% in this study (10 out of 32 participants) was considerably higher than that of 10% in previous studies that adopted LCD monitors [26,38], which is also believed to be attributable to the employment of transparent HUD as the use of transparent display generally weakens SSVEP responses [10]. It is expected that the classification accuracy and illiteracy rate can be further improved by reducing the transparency and increasing the contrast of the visual stimuli using stronger light projectors. Increasing the analysis window size from 2.5 s to longer periods (e.g., 3.5 s) can also be a solution to increase the overall BCI performance [10]; however, optimal window size should be carefully determined considering the convenience of users, because a longer detection time may increase their inconvenience. The development of new algorithms and visual stimuli to increase BCI classification accuracy may also improve the practicality of the proposed in-car environment control system.

Compared to traditional in-car environment control systems that use voice or gesture recognition [39], the proposed BCI-based system offers several advantages. Speech control systems often struggle in noisy environments or when passengers are conversing, whereas BCI systems are less affected by ambient conditions. Unlike gesture-based systems, a BCI system is potentially safer as it allows the driver to maintain control without needing to free their hands for interaction. Although speech or gesture control might be more precise than BCI-based systems, the latter has significant potential. This is particularly true when combined with other BCI applications like drowsiness detection and emotion recognition systems [40,41].

Because the proposed in-car environment control system was evaluated using a driving simulator, further study is needed to investigate the feasibility of the proposed system in a real car environment during actual driving. Although an experiment in a simulated driving environment is a simple, inexpensive, and safe alternative, further evaluations are required to investigate the various potential problems that may occur in real car driving situations. In actual driving conditions, the HUD’s visibility could be compromised due to changing light conditions, weather variations, and transitions between day and night. Since SSVEP depends on visual stimuli, these environmental factors might affect its accuracy. However, advancements in glass display technologies and augmented reality are anticipated to mitigate these challenges. Furthermore, the use of a wearable EEG device instead of a bulky and expensive research-grade recording system will lead to the development of practical BCI applications for in-car environment control. Recently, Mercedes-Benz announced a similar BCI-based concept car that presented visual stimuli on the dashboard [42], implying that car control using BCI is not a preposterous attempt but a promising field that has huge potential to develop into an essential technology to significantly improve our quality of life.

## 6. Conclusions

In this study, we developed an in-car environment control system utilizing an SSVEP-based BCI. This system enables the driver to control the environment inside the car simply by focusing on visual stimuli displayed on the HUD. We evaluated the performance of our proposed system in a realistic driving simulator and compared it to the traditional manual button-press interface. The results showed that the SSVEP-based BCI system had shorter response times and higher rates of responding to obstacles. These findings suggest that the BCI system could reduce the likelihood of car accidents, thereby enhancing driver safety.

## Figures and Tables

**Table 1:** Four Commands for In-Car Environment Control.

Commands			
Turn on Heated Seat	Turn on Seat Ventilation	Temperature Up	Temperature Down

## Data Availability

The data presented in this study are available on request from the corresponding author.
